# Increasing confidence and changing behaviors in primary care providers engaged in genetic counselling

**DOI:** 10.1186/s12909-017-0982-4

**Published:** 2017-09-13

**Authors:** Michael S. Wilkes, Frank C. Day, Tonya L. Fancher, Haley McDermott, Erik Lehman, Robert A. Bell, Michael J. Green

**Affiliations:** 1 School of Medicine, Office of the Dean, University of California, One Shields Avenue, Davis, CA 95616 USA; 2 Departments of Humanities and Medicine, Penn State College of Medicine, 500 University Drive, Hershey, PA 17033 USA; 3 Department of Internal Medicine, Division of General Medicine, University of California, Davis, Sacramento, CA 95817 USA; 4 Department of Emergency Medicine, University of California, Los Angeles, CA 90095 USA; 5 School of Medicine, University of California, Davis, CA 95616 USA; 6 Department of Communication, Department of Public Health Sciences, University of California, Davis, CA 95616 USA

**Keywords:** inherited breast cancer, physician training, BRCA, genetic counseling, genetic testing, shared decision-making

## Abstract

**Background:**

Screening and counseling for genetic conditions is an increasingly important part of primary care practice, particularly given the paucity of genetic counselors in the United States. However, primary care physicians (PCPs) often have an inadequate understanding of evidence-based screening; communication approaches that encourage shared decision-making; ethical, legal, and social implication (ELSI) issues related to screening for genetic mutations; and the basics of clinical genetics. This study explored whether an interactive, web-based genetics curriculum directed at PCPs in non-academic primary care settings was superior at changing practice knowledge, attitudes, and behaviors when compared to a traditional educational approach, particularly when discussing common genetic conditions.

**Methods:**

One hundred twenty one PCPs in California and Pennsylvania physician practices were randomized to either an Intervention Group (IG) or Control Group (CG). IG physicians completed a 6 h interactive web-based curriculum covering communication skills, basics of genetic testing, risk assessment, ELSI issues and practice behaviors. CG physicians were provided with a traditional approach to Continuing Medical Education (CME) (clinical review articles) offering equivalent information.

**Results:**

PCPs in the Intervention Group showed greater increases in knowledge compared to the Control Group. Intervention PCPs were also more satisfied with the educational materials, and more confident in their genetics knowledge and skills compared to those receiving traditional CME materials. Intervention PCPs felt that the web-based curriculum covered medical management, genetics, and ELSI issues significantly better than did the Control Group, and in comparison with traditional curricula. The Intervention Group felt the online tools offered several advantages, and engaged in better shared decision making with standardized patients, however, there was no difference in behavior change between groups with regard to increases in ELSI discussions between PCPs and patients.

**Conclusion:**

While our intervention was deemed more enjoyable, demonstrated significant factual learning and retention, and increased shared decision making practices, there were few differences in behavior changes around ELSI discussions. Unfortunately, barriers to implementing behavior change in clinical genetics is not unique to our intervention. Perhaps the missing element is that busy physicians need systems-level support to engage in meaningful discussions around genetics issues. The next step in promoting active engagement between doctors and patients may be to put into place the tools needed for PCPs to easily access the materials they need at the point-of-care to engage in joint discussions around clinical genetics.

## Background

Screening and counseling for genetic conditions is an increasingly important part of primary care practice, particularly given the paucity of genetic counselors in the United States [1] Discussions about genetic risk-assessment increase patients’ knowledge of genetics, improve the accuracy of their perceived personal risk, and reduce their psychological distress [2]. However, primary care physicians often have an inadequate understanding with regard to evidence-based screening for genetic issues, and a lack of confidence in communicating about shared decision-making, and about legal, ethical, and social issues related to screening for genetic mutations and the basics of clinical genetics [3–7]. Increasing clinician knowledge and confidence in these areas can be challenging. While genetics is a rapidly expanding field, the majority of PCPs are not instructed on these issues during their medical training, and there are few non-industry sponsored education opportunities available to learn the material after the fact. When there is poor physician communication and knowledge, or when the primary care physician (PCP) is unprepared to engage in shared decision-making, the result can be poor medical care – either unnecessary and expensive over testing or inappropriate and potentially dangerous under testing. The result is both an underutilization of genetic counseling, failure to test patients most at risk [4, 8] and an overutilization of testing for those at low risk [9]. When counseling is offered, PCPs often find the task of communicating genetic risks to their patients challenging and give disproportionate attention to screening advantages over disadvantages [10]. Meanwhile, many patients in the U.S. believe that general practitioners are not competent to handle queries about genetic conditions adequately [11]. Thus, there is a clear need for interventions that teach risk assessment [12] and improve provider confidence and adherence to evidence-based genetic counseling and testing recommendations [9, 13].

Evaluations of genetic education material, videos, decision aids, and software programs geared towards the patient have been reported elsewhere [14–19], and several interventions aimed at using interactive curriculum to increase clinician knowledge and attitudes around genetics have been shown to be successful [20, 21]. However few studies have sought to improve physician knowledge, attitudes, communication skills, *and* measured clinician behavior change following the intervention [21]. Thus, the present study explored whether the development and evaluation of an interactive, web-based genetics curriculum directed at PCPs in non-academic primary care settings was superior at changing not only knowledge, confidence, and attitudes with regard to genetic communication, but also was superior in eliciting desired practice behaviors compared with traditional educational approaches. Elsewhere, we have reported on the metrics and outcomes of the practice behavior [22]. In this paper, we focus on the curriculum’s collective impact on clinician knowledge, attitudes, shared decision making practices, and behaviors around genetic issues, and discuss the implications for genetics focused educational interventions moving forward.

## Methods

In this study, 121 California and Pennsylvania PCPs were randomized to either an Intervention Group or Control Group. Physicians in the Intervention Group completed a 6 h interactive web-based curriculum covering information about genetic testing, risk assessment, practice behaviors, attitudes, and communication skills. Physicians in the Control Group were provided with a traditional approach to Continuing Medical Education (CME) (clinical review articles) offering equivalent information. Here we report the effects of the intervention on physicians’ knowledge, attitudes, and intended behaviors.

### Setting and participants

**Fig. 1:**
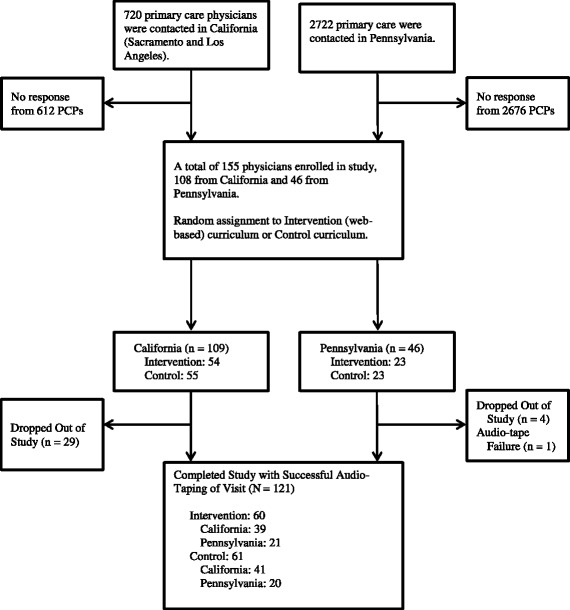
Study Flow Diagram

The study took place in two regions of California (Northern California in the greater Sacramento area and in Southern California in the Los Angeles region) and in central Pennsylvania. PCPs were eligible to participate if they were a Medical Doctor (MD) or Doctor of Osteopathy (DO), a family physician or a general internist, English speaking, did not practice full time in a university setting, and had internet and e-mail access. In California, a list of PCPs was compiled through an internet search of the California Medical Board. Physicians were then sent information about the study through faxes and flyers. Colleagues at clinics in two large health systems made recruitment appeals on our behalf. In Pennsylvania, PCPs were identified via the Pennsylvania Area Health Education Center, which sent personalized letters of invitation, recruitment flyers, and business reply postcards to prospective participants. Using a purchased mailing list, the Pennsylvania State University (PSU) team sent recruitment materials to PCP’s around the state. Participation follows the flow diagram (Fig. 1). Participants were offered 6 units of CME credit and a payment of $250 upon completion of study activities; credit and payment were not affected by individual performance during the standardized patient assessment exercise.

### Design and procedure overview

Study procedures were approved by Institutional Review Boards (IRB) at the University of California, Davis and Pennsylvania State University. Intervention Group (*n* = 60) and Control Group (*n* = 61) PCPs were instructed how to access the curricula learning materials and asked to complete all materials within 60 days. Curricula could be accessed from any web-enabled computer with a moderate speed internet connection.

Upon enrollment, physicians participated in a pre-intervention objective structured video exercise (OSVE) described further below, filled out an online pre-visit questionnaire, completed the educational curriculum, took an online post-visit survey, and finally completed a post-intervention OSVE. After finishing the curriculum, a standardized patient (SP) visit was scheduled. PCPs were only told the SP would be used to evaluate the curriculum; no mention was made of the clinical condition (breast cancer) that would be presented to them. SP visits took place an average of 5 weeks after completion of the curriculum, and were audio-taped, transcribed, coded, and evaluated.

### Intervention and control group curricula

#### Intervention group

Using Kern’s curricular development model [23], a group of 20 consultants with expertise in medical genetics, ethics, law, medical anthropology, clinical medicine, educational assessment, and educational technology from six institutions developed curricular content [24]. Essential ELSI (ethical, legal, and social issues) genetics competencies were identified from organizational recommendations [25] and published reports [26]. Cases illustrated common genetic conditions likely to be encountered by PCPs, and each was linked to competencies/learning-objectives. Intervention Group physicians completed a 6 h interactive web-based curriculum (eDoctoring found at http://edoc.ucdavis.edu/Public_site/) covering information about genetic testing, risk assessment, practice behaviors, and communication skills. The curriculum featured four clinical patient cases (breast cancer, cystic fibrosis, Huntington’s disease, and venous thromboembolism). Each case took 20–30 min to complete and contained multiple video-vignettes (e.g. interactions between patients and physicians) to highlight tensions and communication approaches. Each video or interactive exercise was followed by a relevant discussion between animated physician cartoon characters (aka avatars). Within each case, learners answered closed and open-ended questions to promote understanding and engagement. Some cases also contained unscripted interviews with real patients or experts to illustrate certain points.

Genetics content that applied to more than one case was developed into four content packages called “tutorials” that ran outside of the cases but within the eDoctoring tool. The required tutorials were chosen based on desired learning outcomes and consisted of: 1) core concepts in genetics, 2) core concepts related to screening and testing for disease, 3) ethics and legal issues related to clinical genetics, and 4) shared decision making. Each tutorial took approximately 20–120 min to complete and contained patient focused videos and interactive text. Each case and tutorial included video-vignettes that modeled physician communication, raised questions requiring application of principles, and provided hyperlinks to additional written and video materials. For intervention group clinicians, completion of all four cases and four tutorials was required before they could move on in the study.

eDoctoring was programmed at the University of Newcastle, UK using Flash and Python, linked to MySQL (an open-source relational database management system (RDBMS)) using Zope (a free and open-source web application server written in the Python programming language) and housed on multiple servers in the UK and US allowing hundreds of learners to be on-line simultaneously. The curriculum was alpha-tested with final year primary care residents to improve flow, consistency and navigation.

#### Control group

Control Group participants read eight review articles from leading journals and information sheets extracted from the National Cancer Institute website [27–35]. These nine resources covered clinical genetics, ethical issues, doctor-patient issues, and clinical reasoning, and were topically similar to the material contained in the Intervention Group. Care was taken to ensure that learning objectives and messaging between intervention and Control Group readings were consistent. Participant access and downloading of the articles was recorded electronically. It was estimated that physicians would need at least 6 h to read all articles. While we cannot say how completely or thoroughly the articles were read by control group clinicians, our tracking system allowed us to see that 86% of participants opened the PDFs. The educational Control Group allowed us to examine the impact of our web-based curriculum relative to a more traditional learning format rather than comparing our tools to untaught physicians, which would have been an easy but meaningless comparison.

### Survey instrument development

Before accessing their respective learning tools, learners in both the intervention and Control Group participated in an online, pre-curricular survey to collect information about demographics (3 items), self-efficacy (28 items), knowledge (43 items), and prior experience (13 items) with ELSI and core genetics. The self-efficacy, knowledge, and attitude/experience items were developed based on the learning objectives and published standards from professional groups. [24, 36] Items were later used to form scales to assess improvement from pre to post testing.

#### Self-efficacy

Self-efficacy items [37] were introduced with the stem, “How confident are you about your current skills…” Learners rated their confidence from “not at all confident” [1] to “extremely confident” [5] in a variety of areas. Twenty general self-efficacy items were classified into 2 domains — self-efficacy for genetically related *clinical skills* (e.g., communication, patient teaching, shared decision-making) and self-efficacy related to *genetics knowledge*. Psychometric item analysis and maximum likelihood factor analysis (with varimax rotation) were then used to assess scale internal consistency and dimensionality. Scale refinement was conducted by using baseline data to develop initial scales and follow-up data for validation. In looking at the overall correlations among items in both domains, we found that by dropping one item from the clinical self-efficacy set, we could improve the overall alpha value. After dropping this item, the Cronbach alpha value for clinical skills self-efficacy was .87, and .76 for genetic knowledge self-efficacy.

#### Knowledge

Knowledge was evaluated using an instrument developed for this study, consisting of 38 true/false and multiple choice items. The questions addressed 6 areas of genetics relevant to primary care providers: 1) breast/ovarian cancer genetics, 2) genetic testing, 3) shared decision making, 4) ethical/legal/social issues, 5) venous thromboembolism, and 6) perinatal/pediatric genetic testing. Eleven items related to knowledge about BRCA genetics and breast/ovarian cancer risk, five items related to steps to be taken prior to ordering a genetic test, six items related to shared decision making, six items related to knowledge about ethics, the law, and confidentiality with regard to genetic testing, five items related to general genetics and venous thromboembolism and five items related to ethics and perinatal/pediatric genetic testing.

In both the Intervention and Control Groups, a knowledge test was administered pre- and post-education, and a change score was calculated based on the percent of correct responses. An overall score was also calculated as a percent of correct responses to all items, and compared pre-post and between groups. The Cronbach alpha value for the overall knowledge scale ranged from to .52 pre intervention to .63 post.

#### Prior experience

We also created scales for questions pertaining to prior genetic experience (3 items) and attitude questions (12 items). To assess *prior experience*, learners were asked about their experience with a number of genetic disorders, their experience with caring for those whose main issue was related to a genetic disorder, their experience interacting with genetic counselors or professionals over the past year, and their experience and behaviors with regard to genetic test ordering over the past year. To assess attitudes, learners were asked to give true or false responses to a series of 14 statements regarding ethical, legal, and social implications of genetic testing. Examination of the relationships between questions placed within these domains highlighted items that were not correlative, and these items were thrown out. Through this process, we obtained measures for the following three domains: experience with genetic counselors, attitudes toward genetic screening, and attitudes toward third party access to genetic profiles. The Cronbach alpha values for these measures were .85, .73, and .91 respectively.

#### Assessment of learning tool

At the completion of the entire web-based curriculum, learners in the Intervention Group were asked their overall assessment of the curriculum including whether they would recommend it to others (1 item), overall perceived quality (1 item) and satisfaction with the curriculum (1 item), comparison to traditional curricula (10 items), and perceived impact of curriculum (3 items). Participants were also asked, “In comparison with traditional curricula, how well does this type of interactive web-based tool meet the following goals…” followed by 10 items on a 3 point scales (1 = worse, 2 = the same; 3 = better). Finally, learners were asked how likely they were to make changes to patient care as a result of the curriculum (2 items), as well as perceived barriers in making changes to patient care (7 items).

### Objective structured video exercise (OSVE)

Because the use of standardized patients (SPs) would have been prohibitively expensive and burdensome, we instead used standardized videos to evaluate physician behavior. We designed six objective structured video exercises (OSVEs) to allow for demonstration of the learner’s clinical thinking, judgment, and intended behaviors in response to illustrative video vignettes that heavily emphasized management and ethical issues in genetics care. The goal of the OSVEs was not instructional; they were used purely as assessment instruments.

Upon initial enrollment, physicians were randomly assigned to either OSVE track 1 or OSVE track 2. The different tracks corresponded to the order in which they were shown six 2 to 4 min OSVEs. In the pre-OSVE assessment, physicians were shown three OSVEs. Set one consisted of OSVE A, B, and C, while set two consisted of OSVE D, E, and F. Each of the six OSVEs depicted a doctor-patient/family interaction surrounding a genetic topic. Physicians were shown the opposite set containing 3 OSVEs as a post-intervention. Pre and post OSVEs were matched for content and clinical management skills required, but the OSVEs were not identical. Thus, physicians either saw A, B, and C pre-intervention and D, E, and F post, or vice versa. In the post test, learners saw the series of videos they had not seen in the pre-test. After each OSVE video (pre and post) participants were asked three questions.

Two hundred and forty audio recordings (120 subjects, pre and post) were transcribed by a professional transcription service. Written transcripts were scored by trained graduate students according to a matrix designed and tested by the principal investigators. This matrix awarded 0, 1, and 2 points each for “key items” present in the transcript. Items in the matrix highlighted areas where the learner may have gained understanding or knowledge from the curriculum. Possible points ranged from 0 to 12, 0 to 13, and 0 to 16 for OSVE 1 (videos A and D), 2 (videos B and E), and 3 (videos C and F) respectively. After transcription, 46 randomly selected transcripts out of the 240 total were scored by a second trained graduate student to determine coding reliability and scoring consistency. Both raters were blinded as to which OSVE was the pre and which was the post. Testing for intra-class correlation statistics showed good reliability between the two coders, with scores no less than 0.69 for the pre-intervention OSVE and 0.70 post-intervention.

### Standardized patients

Due to resource limitations, just one of the four medical conditions, breast cancer, was portrayed by an SP. Each physician was visited by one of five trained SPs playing the identical role of “Catherine Douglas,” a woman at risk for inherited breast cancer. Standardized patients were intensively trained on this character and in preparation for the visit via videos and in-person training sessions. SPs were given extensive background on their character, family history, and previous medical history. No medical knowledge of genetics beyond what an informed patient would need to know about breast cancer was provided. Standardized patients were also trained to ask specific questions about the condition and its implications during the encounter. Because of the relative rarity of the medical condition, SP visits were announced [38–41] i.e. physicians knew the woman was a SP but the PCPs did not know until the time of the encounter the reason for Catherine’s visit to the doctor. All encounters were audio recorded and transcribed verbatim. Details of the standardized patient transcript analysis have been reported elsewhere. [22] After the interview, SPs evaluated the physicians on 10 items related to communication style and shared decision making, using a five point Likert scale.

### Analysis

All analyses were performed using SAS Software version 9.3 (SAS Institute, Cary, NC). Before conducting inferential analyses, all variables were summarized with descriptive statistics using percentages for categorical variables and means, medians, and standard deviations for continuous variables to check for errors or outliers. Histograms were also plotted to check the overall distribution of continuous variables. For categorical variables measured only pre-intervention or post-intervention, comparisons were made by Intervention Groups using a Chi-square test, and an exact version of this test was substituted when small cell counts violated the usual assumptions of the asymptotic test. A Wilcoxon Rank Sum test was used to test for differences between Intervention Groups in continuous or ordinal variables measured only pre-intervention or post-intervention given that the distributions of the variables were not normal enough to warrant a Two-sample t-test. The distributions of continuous outcome variables were assessed for normality using histograms, box plots, and normality plots. For ordinal Likert scale outcome variables, nonparametric methods such as the Wilcoxon Rank Sum or Signed Rank tests, which do not require a normal distribution, were used instead.

Changes from pre-intervention to post-intervention in knowledge, self-efficacy, and OSVE scores were compared within and between Intervention Groups using two methods of analysis depending on the distribution of the outcome variable. For outcome variables having an approximately normal distribution, a linear mixed effects model was applied that accounts for the correlation between repeated measures made on the same study participant. This model included factors for the Intervention Group, the study visit, and the interaction between those two factors and allowed all of the pertinent within and between group comparisons to be made. It also made possible the inclusion of all of the outcome data measured at each visit regardless of whether a study participant had complete data at both time points. For outcome variables that had skewed distributions, a Wilcoxon Signed Rank test was used to test for a significant difference from pre to post within each Intervention Group while a Wilcoxon Rank Sum test was used to test for a significant difference between the Intervention Groups in the change from pre to post. Unfortunately this analysis did require complete data with the change being the outcome variable used in the analysis. However, there was a minimum amount of missing data and only one outcome variable, “attitudes toward third party access to genetic profiles,” required this type of analysis.

## Results

Physicians were primarily male, white, non-Hispanic, and middle aged (Table 1). There were no significant differences between the Intervention and Control Groups with regard to demographics, years of practice, or experience with inherited breast cancer. We additionally found no significant region × study group interaction on the study variables, allowing for aggregation of data from the three regions.

**Table 1:** Physician Characteristics by Study Group

Characteristic	Control (*n* = 61)	Intervention (*n* = 60)	Combined (*n* = 121)	*P* ^a^
Age, mean (SD)	48.7 (9.8)	49.1 (10.6)	48.9 (10.1)	0.81
Years since graduation from medical school mean (SD)	21.7 (9.7)	21.3 (12.3)	21.5 (11.0)	0.86
Female, %	34.4	46.7	40.5	0.20
White race, %	67.2	70.0	68.6	0.85
Hispanic, %	4.9	8.3	6.6	0.49
Those with clinical experience managing inherited breast cancer, %	49.2	53.3	51.2	0.72

^a^The significance of mean differences between study groups were examined with t-tests. Differences for the categorical demographic and experience variables were examined with Fisher’s exact test. Nonsignificant differences are suggestive of successful randomization of participants to the two study arms

### Knowledge

Intervention effects on the knowledge measures are reported in Table 2. In both groups, *knowledge about BRCA genetics and breast/ovarian cancer risk* (as measured by the percentage of correct responses) increased significantly (*p* < 0.01) after education (Intervention: 54.7 --> 74.3; Control: 53.1 --> 63.1). The increase in knowledge was significantly greater (*p* < 0.01) in the web Intervention Group (Δ = 19.6) than in the PDF Control Group (Δ = 10.0).

**Table 2:** Group comparisons for knowledge measures at PRE and POST

Variable	Pre		Post		Change (Post-Pre)		
	N	Mean (95% CI)	N	Mean (95% CI)	Mean (95% CI)	Within *P*-value	Between *P*-value
Attitude toward genetic screening (higher=more agreeable, 1-5)*							
Control	67	3.3 (3.1, 3.4)	61	3.4 (3.2, 3.5)	0.1 (0.0, 0.3)	0.17	<0.01
Research	68	3.1 (2.9, 3.3)	61	2.9 (2.7, 3.0)	-0.2 (-0.4, -0.1)	<0.01	
Clinical skills self-efficacy (higher=more confident, 1-5)*							
Control	67	3.0 (2.9, 3.2)	61	3.6 (3.5, 3.7)	0.6 (0.4, 0.7)	<0.01	0.02
Research	68	3.0 (2.8, 3.1)	61	3.8 (3.6, 3.9)	0.8 (0.6, 0.9)	<0.01	
Genetic knowledge (higher=more knowledge, 1-5)*							
Control	67	2.5 (2.4, 2.6)	61	3.3 (3.1, 3.4)	0.8 (0.6, 0.9)	<0.01	0.02
Research	68	2.4 (2.3, 2.6)	61	3.4 (3.3, 3.6)	1.0 (0.9, 1.1)	<0.01	
Attitudes toward third party access to genetic profiles (higher=more agreeable, 1-5)+							
Control	67	1.5 (1.3, 1.6)	61	1.5 (1.3, 1.7)	0.1 (-0.2, 0.3)	0.48	0.64
Research	68	1.4 (1.2, 1.5)	61	1.4 (1.2, 1.5)	0.0 (-0.1, 0.1)	1.0	

* Linear mixed effects model

+ Signed Rank tests for within group comparisons, Wilcoxon Rank Sum test for between group comparison

In the Intervention Group, *knowledge about genetic test ordering* increased significantly (*p* < 0.01) after education (67.9➔80.0), but there was no increase in knowledge in the Control Group (64.8➔64.5). The increase in knowledge was significantly greater (*p* < 0.01) in the Intervention Group (Δ = 12.1) than in the Control Group (Δ = −0.3). There was no increase in *knowledge about shared decision making* in either group. In both groups, knowledge about *ethics, the law, and confidentiality* related to genetic testing increased significantly after education (intervention: 65.9➔81.6, *p* < 0.01; control: 64.4➔70.2, *p* = 0.03). However, the increase in knowledge was significantly greater (*p* < 0.01) in the Intervention Group (Δ = 15.7) than in the Control Group (Δ = 5.8).

In the Intervention Group, knowledge related to *general genetics and venous thromboembolism* increased significantly after education (65.5➔86.9, *p* < 0.01), but there was no increase in knowledge in the Control Group (63.6➔68.3, *p* = 0.12). The increase in knowledge was significantly greater (*p* < 0.01) in the Intervention Group (Δ = 21.5) than in the Control Group (Δ = 4.7). In both groups, knowledge related to ethics and perinatal/pediatric genetic testing increased significantly (*p* < 0.01) after education (Intervention: 70.3➔87.8; Control: 65.8➔76.1). The increase in knowledge was not different between groups (*p* = 0.08).

A cumulative knowledge score based on percent correct of the 38 above items was calculated. In both groups, knowledge increased significantly (*p* < 0.01) after education (Intervention: 62.1➔75.9; Control: 59.1➔65.5). However, the increase in knowledge was significantly greater (*p* < 0.01) in the Intervention Group (Δ = 13.8) than in the Control Group (Δ = 6.3).

### Self-efficacy

After participation, PCPs’ self-efficacy improved in both groups for both domains (*P* < 0.01), with modest but significantly higher improvement in the Intervention Group (Table 3). PCPs were initially “somewhat” confident with their ELSI-genetics skills, improving to “more” confident after curricular participation. PCPs were initially less confident about their ELSI-genetics knowledge, improving to “somewhat” to “more” confident after curricular participation.

**Table 3:** Changes in primary care providers’ self-efficacy and attitudes before and after intervention and control curricular participation, in four domains

Variable	Pretest		Post-test		Change	*P* value	
	N	Mean (95% CI)	N	Mean (95% CI)	Mean (95% CI)	Within	Between
Knowledge about BRCA genetics and breast/ovarian cancer risk (11 items) % correct							
Control	67	53.1 (49.5, 56.6)	61	63.1 (59.4, 66.8)	10.0 (5.8, 14.2)	<0.01	<0.01
Research	66	54.7 (51.1, 58.3)	61	74.3 (70.5, 78.0)	19.6 (15.4, 23.8)	<0.01	
Knowledge about genetic testing (5 items) % correct							
Control	67	64.8 (61.2, 68.3)	61	64.5 (60.8, 68.2)	-0.3 (-3.9, 3.4)	0.89	<0.01
Research	66	67.9 (64.3, 71.4)	61	80.0 (76.3, 83.7)	12.1 (8.4, 15.8)	<0.01	
Knowledge about breast cancer overall % correct							
Control	67	56.7 (54.0, 59.5)	61	63.5 (60.7, 66.4)	6.8 (3.8, 9.8)	<0.01	<0.01
Research	66	58.8 (56.0, 61.6)	61	76.1 (73.2, 78.9)	17.2 (14.2, 20.2)	<0.01	
Knowledge about Shared Decision Making % correct							
Control	67	63.6 (58.8, 68.4)	61	67.8 (63.7, 71.9)	4.2 (-0.9, 9.3)	0.11	0.84
Research	66	67.9 (63.1, 72.7)	61	71.4 (67.3, 75.5)	3.4 (-1.7, 8.6)	0.19	
Knowledge about ethics, law, confidentiality % correct							
Control	66	64.4 (59.5, 69.3)	61	70.2 (66.0, 74.4)	5.8 (0.7, 10.9)	0.03	<0.01
Research	66	65.9 (61.0, 70.8)	61	81.6 (77.4, 85.9)	15.7 (10.6, 20.8)	<0.01	
Knowledge about VTE % correct							
Control	66	63.6 (58.6, 68.7)	61	68.3 (64.0, 72.6)	4.7 (-1.3, 10.6)	0.12	<0.01
Research	66	65.5 (60.4, 70.5)	61	86.9 (82.6, 91.2)	21.5 (15.6, 27.4)	<0.01	
Knowledge about ethics and perinatal/pediatric genetic testing % correct							
Control	66	65.8 (60.9, 70.6)	61	76.1 (71.5, 80.7)	10.4 (4.6, 16.1)	<0.01	0.08
Research	66	70.3 (65.5, 75.1)	61	87.8 (83.3, 92.4)	17.5 (11.8, 23.2)	<0.01	
Total Knowledge % correct							
Control	66	59.1 (56.9, 61.4)	61	65.5 (63.6, 67.4)	6.3 (4.4, 8.3)	<0.01	<0.01
Research	66	62.1 (59.9, 64.4)	61	75.9 (74.0, 77.8)	13.8 (11.9, 15.8)	<0.01	

* Linear mixed effects model

### Attitudes

PCPs in both groups were initially neutral with regard to the value of genetic profiling by individuals. After participation, PCPs in the Intervention Group tended to *disagree* that individuals should be genetically profiled, and PCPs in the Control Group remained neutral. PCPs in both groups initially indicated that insurers and employers should not have access to an individual’s genetic profile, and neither group changed this opinion after the curriculum (Table 3).

### OSVE

The OSVE assessment showed that overall the intervention and Control Group both improved significantly from pre to post OSVE; neither group improved more than the other (Table 4).

**Table 4:** Group comparisons for OSVE measured at PRE and POST

Variable	Pretest		Post-test		Change	Within *P*-value	Between *P*-value
	*N*	Mean (95% CI)	*N*	Mean (95% CI)	Mean (95% CI)		
OSVE 1 Total Score (0-12)							
Control	60	3.4 (3.0, 4.0)	59	4.0 (3.6, 4.6)	0.6 (0.0, 1.2)	0.05	0.56
Intervention	58	3.2 (2.6, 3.6)	59	4.0 (3.6, 4.6)	0.8 (0.2, 1.6)	<0.01	
OSVE 2 Total Score (0-13)							
Control	61	3.8 (3.4, 4.4)	61	5.2 (4.6, 5.6)	1.2 (0.6, 1.8)	<0.01	0.99
Intervention	58	4.2 (3.6, 4.6)	59	5.4 (4.8, 6.0)	1.2 (0.6, 2.0)	<0.01	
OSVE 3 Total Score (0-16)							
Control	60	4.0 (3.4, 4.6)	61	4.6 (4.0, 5.2)	0.6 (-0.2, 1.2)	0.17	0.88
Intervention	59	4.4 (3.8, 4.8)	59	4.8 (4.2, 5.4)	0.4 (-0.4, 1.2)	0.25	
OSVE Total Score (0-40)							
Control	59	11.2 (10.0, 12.4)	59	13.8 (12.6, 15.0)	2.6 (1.2, 3.8)	<0.01	0.69
Intervention	55	11.4 (10.0, 12.6)	57	14.2 (13.0, 15.4)	3.0 (1.6, 4.4)	<0.01	

*Linear mixed effects model

### Practice behaviors (transcript analysis of SP visits)

Based on review of SP transcripts, we found that the average physician in this study asked just 20% of the family and personal history questions that would be appropriate in this setting, a finding consistent with other studies [42, 43]. Physicians also fell short in their counseling about the implications of positive test results, focusing heavily on surgical options and neglecting to explore familial implications, emotional impacts, or social support, though each of these is an essential component of the genetic counseling process [44–47]. Furthermore, though considerable attention was devoted to ELSI issues in the web-based and control curriculum, only half of the participants ever talked with the SP about medical record confidentiality, insurance discrimination, and federal legal protections or the impact of testing on relatives. Even fewer examined employment discrimination concerns, and none explored the important issue of social stigma [45]. However, intervention doctors were more likely to engage in key shared decision-making behaviors, such as suggesting the choice of testing was up to the patient (control doctors were more likely to recommend the test), encouraging a discussion with a genetic counsellor, and asking about prostate and ovarian cancer [22].

SPs also provided a post-encounter evaluation via a 10 item, five-point Likert scale questionnaire focused on clinician communication and shared decision making skills. While this type of evaluation was more subjective than the transcript analysis, SPs were blind as to which group each physician was in. Though not statistically significant, there was an overall trend by intervention physicians toward higher ratings (strongly agree plus agree) including communication skills (97% vs 91%), “offering me different options for care” (95% vs 90%), “encouraged me to ask questions” (77 vs 73), “I felt the doctor listened to me” (92% vs 80%), “I felt the doctor took enough time with me” (91% vs 88%). There were no areas where the Control Group performed better.

### Attitude toward tools

#### Satisfaction with tools

The Control Group reported completing on average 87% of the curriculum; the intervention reported completing 92%. Compared to the Control Group, intervention PCPs were significantly more likely to rate the curriculum highly (*p* < .0001). They were also significantly more likely to recommend the curriculum to colleagues (*p* < .0001). When asked about the impact of what they learned, the Intervention Group rated several areas significantly higher than for the Control Group: the acquisition of ELSI (ethical legal, and social) knowledge (*p* < 0.0002), confidence in caring for people with genetic conditions (*p* < .02), communication issues around genetics (*p* < 0.01) and ability to care for people with genetic disorders (*p* < 0.01). Intervention PCPs (78%) reported that they would prefer to participate in this web-based program compared with the option of reading materials (either online or in paper) or attending a CME class (results not presented in tabular form).

#### Advantages/disadvantages of the tool

Intervention Group PCPs in their end-of-curriculum survey responses rated learning related to medical management, genetic core concepts, and ELSI issues significantly higher than the Control Group. In comparison with traditional curricula, Intervention Group PCPs felt that their curriculum offered several advantages including being engaging, addressing communication styles, and providing information of clinical relevance (Table 5). There was no difference between groups in their estimated likelihood that clinical changes would result from the CME learning activity. Participants were also asked about perceived barriers to implementing learned materials (cost, lack of time for patient care, lack of administrative support, insurance coverage/ reimbursement issues, patient compliance issues, uncertainty about professional consensus) to implementing learned materials. While both groups perceived significant barriers, there were no differences between groups.

**Table 5:** Comparison of CME medical education curriculum with interactive web-based education

“In comparison with traditional CME medical education curricula, how well does this type of interactive web-based education meet the following goals?”	Worse, n (%)		About the same, n (%)		Better, n (%)tter, n (%)		*P*
	CNTL	INV	CNTL	INV	CNTL	INV	
Fits into your schedule	4(6.56)	4(6.56)	12(19.67)	13(21.31)	45(73.77)	44(72.13)	NS
Demonstrates good/bad communication styles	14(22.95)	0(0.00)	35(57.38)	17(27.87)	12(19.67)	44(72.13)	0.0001
Stimulates self-directed learning	7(11.48)	2(3.28)	28(45.90)	22(36.07)	26(42.62)	37(60.66)	0.0271
Provide learning flexibility in time/place	0(0.00)	1(1.64)	11(18.03)	9(14.75)	50(81.97)	51(83.61)	NS
Provides opportunities to explore additional clinical content	9(14.75)	1(1.64)	40(65.57)	31(50.82)	12(19.67)	29(47.54)	0.0002
Answers practical questions about the content	20(32.79)	9(14.75)	30(49.18)	32(52.46)	11(18.03)	20(32.79)	0.0102
Engages you in the content	13(21.31)	2(3.28)	28(45.90)	13(21.31)	20(32.79)	46(75.41)	0.0001
Help you utilize content in the care of patients	8(13.11)	1(1.64)	37(60.66)	28(45.90)	16(26.23)	32(52.46)	0.0008
Facilitates long-term retention of knowledge	12(19.67	2(3.28)	40(65.57)	39(63.93)	9(14.75)	20(32.79)	0.0013
Stimulates self-reflection about your skills	11(18.03)	1(1.64)	28(45.90)	26(42.62)	22(36.07)	34(55.74)	0.0050

## Discussion

With the rapid proliferation of genetic tests, the need for genetic counseling services will continue to grow. Alongside trained genetic counselors, informed primary care physicians who are able to engage in shared decision-making and evidence-based test ordering will be crucial in responding to this growth. However, many primary care physicians have an insufficient understanding of the basics of clinical genetics, evidence-based screening, communication approaches that encourage shared decision-making, and awareness of ethical and social issues related to screening for genetic mutations [4]. Thus, there is a need for effective targeted interventions that improve physician competencies with regard to genetic testing, counseling, and communication. In this study, we found that compared to standard curricular materials, an interactive web-based CME curriculum was more effective at increasing physician knowledge around genetic testing, and improving shared decision making behaviors, though it had a small effect on attitudes and minimally impacted clinical behaviors around ethical, legal, and social discussions around genetic testing.

Overall, PCPs in the Intervention Group showed greater increases in knowledge compared to the Control Group. Intervention PCPs were also more satisfied with the educational materials, and more confident in their ELSI genetics knowledge and skills compared to those receiving traditional CME materials. Intervention clinicians were also significantly better at implementing shared decision making. While both groups received information around shared decision making in curricular materials, and while there was no difference between the groups with regard to knowledge of shared decision making, intervention doctors were more likely to engage in shared decision-making in standardized patient visits as determined by transcript analysis and SPs rated them higher on a 10 item questionnaire around communication and shared decision making skills. This discrepancy between knowledge and application has important implications for shared decision making and for our curriculum. The interactive tool required interaction and engagement with clinical questions, observation of communication skills, and discussions of barriers to effective genetic testing. This may imply that while the fund of knowledge around SDM may be common and well-accepted among PCPs, implementation may require a demonstrative curriculum such as ours which allowed PCPs to see various models of implementation in multiple clinical situations.

In general, based on SP ratings and review of SP-physician transcripts, the intervention’s impact on practice behaviors was only minimally better than the Control Group. Physicians in both groups infrequently performed key counseling behaviors related to inherited breast cancer (family history of cancer, questions about onset of breast cancer in close relatives, or discussion of risks and benefit of screening either by genetic testing or imaging), regardless of whether they had completed the web-based training or read traditional clinical reviews. Though the study was not designed to determine why such behaviors were or were not performed, the results raise the question of what additional opportunities for reinforcement, application, or prompting would be needed to facilitate behavior change. Our findings suggest that increasing knowledge and confidence do not necessarily translate to actions in the clinical setting, and further research is warranted to help identify strategies that impact clinical interactions around genetic diseases. This finding is not unique, as additional studies looking to impact behavior have often proved ineffective or not sustainable over time [21].

There were several limitations that deserve mentioning**.** A number of physicians invited to participate did not respond to the invitation, leading to the possibility of a selection bias wherein motivated and interested physicians were overrepresented. The effect of such a selection bias would be to overstate the true level of provider ability in the domain studied. Second, the SPs were announced, and although physicians had no advanced knowledge of the clinical presentation, they did know that they were being evaluated. They may, therefore, have exhibited a higher level of clinical skills than would have been observed had the SPs been unannounced [39]. Third, we examined just one genetic condition; findings may not generalize to other conditions. Finally, the study was carried out in only two states.

## Conclusion

This trial evaluated a novel web-based educational intervention aimed at improving communication skills, addressing ELSI issues, and enhancing shared decision-making. To practice high quality medicine, it is necessary to have an ongoing commitment to acquiring new information but as it turns out, this is not sufficient. Traditional methods of continuing medical education have relied heavily upon large group dissemination of information such as lectures and/or readings. While our intervention was deemed more enjoyable, demonstrated significant factual learning and retention, and improved implementation of shared decision making, there were few differences in ELSI behaviors between the intervention and control groups. There is a growing literature that suggests that behavior change requires not only readiness to change by the physician (providing the knowledge and background and demonstrating “best practice”) but also behavioral prompts to increase uptake such as clinical decision support, just-in-time information availability, clinician reimbursement for genetics-related activities, and prompting by an educated consumer [5, 21, 48, 49]. Perhaps the missing element, therefore, is that busy physicians need system-level support to engage in meaningful discussions around the ethical, legal, and social implications of genetic testing. In this study as well as others, knowledge and attitudes around genetic issues have been shown to increase with interactive educational interventions. However, we have confirmed, like others, that brief educational interventions are unlikely to impact clinician behaviors around genetic interactions long-term [48]. Thus, we concur with other studies that suggest that measuring changes in knowledge and attitudes should not be the prime objective of educational interventions around genetics moving forward [21]. Instead, measuring ethical, legal, and social awareness of genetic issues, as well as the ability to locate and utilize that genetic information when needed- may be a better aim for future interventions. Thus, the next step in promoting active engagement between doctors and patients may be to put into place the tools and infrastructure that allow clinicians to easily access evidence-based information during the encounter, preparing physicians to engage in the joint discussions around genetic issues that patients need.
